# Electroencephalography spectral edge frequency and suppression rate-guided sedation in patients with COVID-19: A randomized controlled trial

**DOI:** 10.3389/fmed.2022.1013430

**Published:** 2022-11-04

**Authors:** Eduardo Tobar, José I. Farías, Verónica Rojas, Antonello Penna, José I. Egaña, Daniela Ponce, Daniela Bravo, Felipe Maldonado, Abraham Gajardo, Rodrigo Gutiérrez

**Affiliations:** ^1^Critical Care Unit, Department of Medicine, Hospital Clínico de la Universidad de Chile, Santiago, Chile; ^2^Centro de Investigación Clínica Avanzada (CICA), Hospital Clínico de la Universidad de Chile, Santiago, Chile; ^3^Department of Anesthesia and Perioperative Medicine, Faculty of Medicine, University of Chile, Santiago, Chile

**Keywords:** sedation, COVID-19, electroencephalogram, bispectral index, suppression rate, spectral edge frequency

## Abstract

**Background:**

Sedation in coronavirus disease 2019 (COVID-19) patients has been identified as a major challenge. We aimed to investigate whether the use of a multiparameter electroencephalogram (EEG) protocol to guide sedation in COVID-19 patients would increase the 30-day mechanical ventilation-free days (VFD).

**Methods:**

We conducted a double-blind randomized clinical trial. We included patients with severe pneumonia due to COVID-19 who required mechanical ventilation (MV) and deep sedation. We randomized to the control (*n* = 25) or multiparameter group (*n* = 25). Sedation in the intervention group was administered following the standard institutional protocols together with a flow chart designed to reduce the propofol administration dose if the EEG suppression rate was over 2% or the spectral edge frequency 95 (SEF95) was below 10 Hz. We performed an intention-to-treat analysis to evaluate our primary outcome (30-day VFD).

**Results:**

There was no difference in VFD at day 30 (median: 11 [IQR 0–20] days in the control group vs. 0 [IQR 0–21] days in the BIS multiparameter group, *p* = 0.87). Among secondary outcomes, we documented a 17% reduction in the total adjusted propofol administered during the first 5 days of the protocol [median: 2.3 (IQR 1.9–2.8) mg/k/h in the control group vs. 1.9(IQR 1.5–2.2) mg/k/h in the MP group, *p* = 0.005]. This was accompanied by a higher average BIS value in the intervention group throughout the treatment period.

**Conclusion:**

A sedation protocol guided by multivariate EEG-derived parameters did not increase the 30-day VFD. However, the intervention led to a reduction in total propofol administration.

## Introduction

Patients infected with the SARS-CoV-2 virus may develop severe pneumonia and acute respiratory distress syndrome (ARDS) requiring mechanical ventilation (MV) (1). They challenge Intensive Care Unit (ICU) teams in terms of ventilatory management and non-ventilatory support (2). Providing an adequate level of sedation in this scenario has arisen as particularly difficult (3, 4).

Deep sedation, even early, impacts hospital outcomes and long-term follow-up (5). Thus, international guidelines promote light sedation in most clinical situations. However, they do not suggest any specific recommendation for ARDS patients, despite they are at an increased risk of receiving deep sedation (6). Besides, COVID-19 patients who are on MV indeed require deep sedation, neuromuscular blocking (NMBs), and prone positioning, with higher use and duration than those previously reported for ARDS (7). It has also been recognized that SARS-CoV-2 may generate neuroinflammation directly (8). Consequently, COVID-19 patients have a higher risk of complications derived from excessive/prolonged sedation (9, 10).

To adequately provide deep sedation and minimize its adverse consequences, it has been suggested to monitor electroencephalographic (EEG) activity through anesthetic depth monitors. Although the evidence supporting their use in the ICU is of low quality, PADIS guidelines, as well as experts’ opinions, suggest such use in patients who require deep sedation together with NMBs (11). Among the available devices, bispectral index (BIS™) monitoring is the most studied (12). A goal suggested for BIS monitoring under surgical general anesthesia is an index value of 40–60 (13), and despite the ICU is a different scenario, similar targets have been adopted for critically ill patients requiring deep sedation (14). However, compelling evidence suggests that the simplification of the EEG signal into one index may be misleading (15, 16). The algorithms that generate these indices may be affected by several factors, such as technical artifacts, the use of other drugs (e.g., ketamine, NMB, or nitrous oxide), or even patient conditions (hypoglycemia or hypothermia) (17, 18). Thus, complementary metrics derived from the EEG together with the raw EEG have been proposed to optimize and personalize the effect of hypnotic on the brain (19, 20). Indeed, some devices, such as BIS Vista™ (Medtronic) monitor, allow the user to visualize in real time other variables derived from the frontal EEG beyond the index, such as the spectral edge frequency (SEF95), the Suppression Ratio (SR) and the Density Spectral Array, which could promote a more intensified and tailored use of EEG to reduce the use of sedatives in the population of patients with ARDS that require deep sedation (21, 22). The potential benefits of diminishing sedative administration may be a reduction in neurological complications such as delirium, but also, in theory, may reduce the amount of drug accumulation in the adipose tissue. For highly lipophilic drugs such as propofol, the apparent distribution volume increases with the length of the continuous infusion due to drug accumulation in slow-equilibrating compartments (23). This is particularly important considering the influence of allometric characteristic in the behavior of fast and slow-equilibrating compartments (24). Ultimately, this leads to a longer time to elimination after the infusion is terminated as the drug distributes back into the central compartment (25). Thus, reducing the time for clearance of the drug from the body may facilitate the weaning process and therefore may reduce the number of days on MV. Therefore, we hypothesized that if we avoid excessive sedation in the acute phase, patients will spend less time in MV.

We conducted a randomized clinical trial in mechanically ventilated COVID-19 patients to evaluate whether deep sedation guided by a protocol based on the intensive use of BIS monitoring parameters (BIS™, SEF95, and SR) allows an increase in ventilator-free days (VFD) at 30 days compared with a control group. Secondly, we aimed to assess whether the proposed protocol would reduce the use of propofol in COVID-19 patients.

## Materials and methods

### Design and participants

This study was approved by the local ethics committee (December 2020, Number 80,320), and written informed consent was obtained from a family member (or legal representative) of every patient included. A double-blind parallel randomized clinical trial was conducted in patients older than 18 years old admitted to the ICU due to severe COVID-19 pneumonia requiring MV. We excluded patients with contraindications to receive propofol or fentanyl, chronic liver disease, child C stage, and end-stage kidney chronic disease. Patients from the COVID-19 Critical Care Units of the “Hospital Clínico de la Universidad Chile” were randomized no later than 48 h after being intubated to either the control or “BIS multiparameter (MP)” group (in a 1:1 ratio) using a simple computer-generated sequence carried out by a researcher not involved in direct patient care. Sequentially numbered sealed opaque envelopes were used. Although nursing personnel were aware of the allocation group, treating physicians, respiratory therapists, patients, and outcome assessor were blinded to the allocation. Besides, non-nursing personnel were not trained to interpret and read the new parameters incorporated in the BIS monitor. The protocol was registered on Clinicaltrials.gov (NCT 04699916) before the first patient enrollment (January 7, 2021), and no changes were made thereafter. The last patient was enrolled on April 27, 2021. Registration name: EEG-based Sedation Protocol for Patients on MV Due to SARS-CoV-2 Pneumonia.

### Interventions

All admitted COVID-19 patients were managed according to the institutional protocol detailed in Supplementary Methods 1, 2. The patients we randomized to either:

*Control group:* To guide sedation, we perform a clinical evaluation with the Sedation-Agitation Scale (SAS) (26). The daily goal for patients with severe COVID-19 pneumonia with MV and PaO_2_:FiO_2_ less than 150 was SAS 1–2 (Supplementary Method 2). Plus, in all patients with an indication for deep sedation with or without infusion of NMBs or prone positioning, we used a BIS™ monitor. Together with the SAS goal, sedatives were delivered to a target of BIS between 40 and 60.

*MP group (intervention):* An intensified MP protocol was developed by the research team. It consisted of the use of the clinical evaluation scale (SAS) and BIS, together with additional EEG-derived parameters (SEF95 and SR), according to a flow chart designed (Supplementary Figure 3). SEF95 is a spectral metric related to the frequency components of the EEG signal. SR is the proportion of time in which the EEG signal was isoelectric in the last 63 s. Both parameters are now displayed on the new BIS Vista™ monitors in real time. Thus, nurses adjusted the propofol doses to maintain SAS 1–2 and BIS 40–60 and to avoid an SR over 2% and an SEF95 lower than 10 Hz. The intervention was designed as a stepped protocol, in which nurses first consider the SAS and the BIS value, and only once the targeted sedation level is achieved (deep sedation), they adjusted the propofol infusion rate to avoid a “too” deep sedation state. The latter was based on optimizing both SR and the SEF95. This protocol was discussed and taught to all the nursing teams in our participating ICUs 1–2 months before starting patients’ enrollment. Considering previous studies in ARDS patients in which they spent between 5 and 7 days in deep sedation and since the protocol was designed to avoid oversedation through this early phase of the disease, we define 5 days for the duration of our intervention (27, 28). This time frame also allowed us to ensure the feasibility for the implementation of this protocol among the nurse teams in a pandemic scenario.

### Data collection

A trained person recorded clinical data from medical and nursing records daily. These were compiled into a coded database that included physiological variables, drugs administered (sedatives, vasoactive drugs), pain and sedation scales. Regarding the outcomes, these patients were also followed up daily during the hospital stay and received subsequent telephone follow-up at 90 days to evaluate mortality. We also extracted the derived parameters from the BIS™ monitors at the end of the study participation.

### Outcomes

Our primary outcome was VFD at day 30. The secondary outcomes were the total administered dose of propofol (adjusted by weight and infusion duration) and fentanyl during the first 5 days, duration (days) and peak dose (μg/kg/min) of norepinephrine during the first 5 days, incidence of suspected adverse events related to propofol infusion, propofol-related infusion syndrome (PRIS) cumulative incidence (first 5 days) (29), non-programmed extubation, delirium incidence, delirium duration, tracheostomy rate, ICU and hospital length of stay, success of the first ventilator weaning trial, and 30-day mortality.

### Statistics and sample size

No reliable data were available at the moment of trial design to allow for an accurate sample size calculation, regarding the potential benefit of intensified BIS monitoring on VFD in patients with ARDS. Therefore, we used data from other studies evaluating different interventions to increase VFD. Previous studies have documented a 20% increase in VFD at 30 days with positive end-expiratory pressure trial interventions (30). We estimated that the BIS multiparameter group would have 18 ± 3 VFD at 30 days compared to 15 ± 3 in the control group. To detect this 20% effect size with a power of 80%, a two-tailed alpha of 0.05, and a 10% drop-out rate, we estimated 25 patients per group. This estimation was performed with G*Power 5.1 Software.

Categorical variables are presented as numbers (percentages) and were compared by Fisher’s exact test. Continuous variables are presented as the mean [standard deviation (SD)] or the median interquartile range (IQR) depending on the data distribution evaluated by the Shapiro–Wilk test; two-group comparisons were made by Student’s *t*-test or Mann–Whitney’s *U*-test, respectively. We follow CONSORT guidelines to report our results (31) (Supplementary Method 4).

The statistical analysis plan is presented in Supplementary Method 5. An intention-to-treat analysis was performed for the primary outcome. We additionally analyzed the primary outcome only in those patients who received propofol for deep sedation, and we detailed the reported causes for propofol suspension. For secondary outcomes, the level of significance was adjusted with the Bonferroni correction. Finally, a *post hoc* exploratory analysis was performed to assess the independent effect of the BIS multiparameter protocol on the use of sedatives adjusting with known potential confounders. For each variable, we performed mixed-effects linear regression, considering the daily accumulated propofol dose as a dependent variable, each patient as a random effect, and the fixed effects (age, sex, fentanyl use, use of prone positioning, and neuromuscular blockade). All analyses were performed in Stata v.14 (Texas, USA) and Prism GraphPad v9.2 (California, USA).

## Results

**FIGURE 1:**
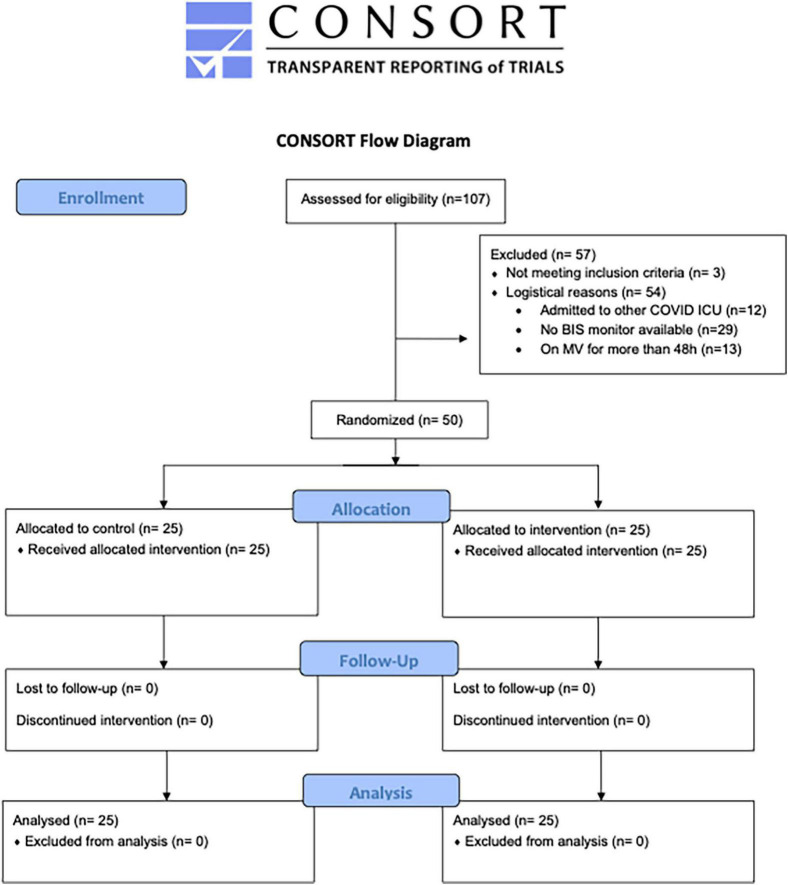
Consort flow chart of the study. COVID, coronavirus disease; ICU, intensive care unit; BIS, bispectral index; MV, mechanical ventilation (31).

The enrollment of patients started in January 2021 and finished in May 2021. We enrolled 25 in each group (Figure 1). Demographics and baseline characteristics are shown in Table 1. The characteristics of analgesia, sedation, neuromuscular blockers, and MV before enrollment are also detailed in Table 1.

**TABLE 1:** Demographics and baseline physiological data.

	Control group (*n* = 25)	Multiparameter protocol (*n* = 25)	*P*-value
**Demographics**			
Age–years, mean (SD)	56.4 (14.9)	56.8 (15.4)	0.46
Sex–female, no (%)	8 (32)	11 (44)	0.56
Body mass index–kg/m^2^, median (IQR)	30.0 (26.4–34.2)	30.8 (26.4–35.5)	0.53
CCI—score, median (IQR)	1 (0–1.5)	0 (0–1)	0.24
Hypertension—yes, no (%)	11 (44)	10 (44)	1.00
Diabetes mellitus—yes, no (%)	8 (32)	6 (24)	0.75
**ICU baseline physiological parameters^a^**			
SAS—score, median (IQR)	1 (1–1)	1 (1–1)	0.65
BPS—score, median (IQR)	3 (3–3)	3 (3–3)	0.31
NMB—yes, no (%)	23 (92)	20 (80)	.23
Fentanyl infusion rate—mcg/k/h, median (IQR)	2.8 (2.2–3.4)	2.9 (2.5–4.4)	0.89
Propofol infusion rate—mg/k/h, median (IQR)	2.5 (2.2–2.8)	2.3 (2.0–2.8)	0.76
Midazolam administration—yes, no (%)	4 (16)	4 (16)	0.50
Midazolam/propofol coadministration—yes, no (%)	2 (8)	3 (12)	0.32
APACHE II—score, median (IQR)	16 (13.5–18.5)	16 (14–18)	0.95
SOFA—score, median (IQR)	6 (4.5–7)	6 (5.5–7)	0.88
pH, median (IQR)	7.36 (7.32–7.41)	7.35 (7.31–7.4)	0.32
Lactate—mg/dL, median (IQR)	1.3 (1–1.5)	1.2 (1–1.4)	0.67
Triglycerides—mg/dL, median (IQR)	281 (202–440)	272 (226–314)	0.86
Time from OIT to randomization—hours, median (IQR)	13 (9.6–16.4)	12.7 (8–16.8)	
PaO_2_/FiO_2_—cmH_2_O, median (IQR)	122 (95–172)	123 (104–184)	0.62
PaCO_2_—cmH_2_O, median (IQR)	36 (32–45)	41 (35–49)	0.31
Tidal volume—mL/kg IBW, median (IQR)	5.9 (5.4–6.1)	6.1 (5.7–6.6)	0.06
Driving pressure—cmH_2_O, median (IQR)	11 (10–12)	10 (8.2–12.8)	0.44
PEEP—cmH_2_O, median (IQR)	10 (8–12)	10 (8–12)	0.25
Plateau pressure—cmH_2_O, median (IQR)	20 (18–22)	21 (19–22.5)	0.17
Prone position—yes, no (%)	15 (60)	12 (48)	0.40

^a^Before randomization.

SD, standard deviation; kg, kilograms; m, meters; CCI, Charlson Comorbidity Index; ICU, intensive care unit; SAS, Sedation Agitation Scale; BPS, Behavioral Pain Scale; NMB, neuromuscular blockade; APACHE II, Acute Physiology and Chronic Health Evaluation, IBW, ideal body weight; OIT, orotracheal intubation; PaO_2_, partial pressure of arterial oxygen; FiO_2_, fraction of inspired oxygen; PaCO_2_, partial pressure of carbon dioxide; PEEP, positive end expiratory pressure.

The primary and secondary outcomes are presented in Table 2. There was no difference in VFD at day 30 [median: 11 (IQR 0–20) days in the control group vs. 0 (IQR 0–21) days in the MP group, *p* = 0.87, Supplementary Figure 8]. Among secondary outcomes, we documented a 17% reduction in the total adjusted propofol administered during the first 5 days of the protocol [median: 2.3 (IQR 1.9–2.8) mg/k/h in the control group vs. 1.9 (IQR 1.5–2.2) mg/k/h in the MP group, *p* = 0.005].

**TABLE 2:** Primary and secondary outcomes.

	Control group (*n* = 25)	Multiparameter group (*n* = 25)	*P*-value
**Primary outcome**			
Ventilator-free days—days, median (IQR)^a^	11.0 (0–20)	0 (0–21)	0.80
**Secondary outcomes^b^**			
1. Total propofol consumption—mg, mean (SD)^c^	17.974 (11.225)	12.182 (8.150)	0.02
2. Total propofol consumption—mg/kg/h, median (IQR)^c^	2.3 (1.9–2.8)	1.9 (1.5–2.2)	0.005
3. Total fentanyl consumption—mcg/kg/h, median (IQR)^c^	2.5 (2.1–3.1)	2.7 (2.3–3.2)	0.38
4. Peak norepinephrine infusion rate—mcg/kg, median (IQR)^b^	0.05 (0.02–0.1)	0.06 (0.02–0.08)	0.95
5. Unprogrammed extubation—yes, no (%)	0 (0)	0 (0)	0.99
6. Delirium incidence—no (%)^a^	9 (36)	13 (52)	0.39
7. Delirium days—days, median (IQR)^a^	0 (0–1)	1 (0–3)	0.25
8. Coma days—days, median (IQR)^a^	14 (8–27)	9 (8–22)	0.28
9. Successful weaning—yes, no (%)^a^	13 (52)	12 (48)	>0.99
10. Tracheostomy—yes, no (%)^a^	44 (11)	32 (8)	0.39
11. PRIS incidence—yes, no (%)^a^	0 (0)	0 (0)	>0.99
12. Hospital and alive free days, days, median (IQR)	43 (0–60)	52 (21–69)	0.56
13. 30-day mortality—yes, no (%)	2 (8)	1 (4)	0.28
14. 90-day mortality—yes, no (%)	5 (20)	2 (8)	0.11

^a^At day 30.

^b^Level of significance adjusted with Bonfenorri’s correction to be 0.005 at day 90.

^c^During the first 5 days after randomization.

**FIGURE 2:**
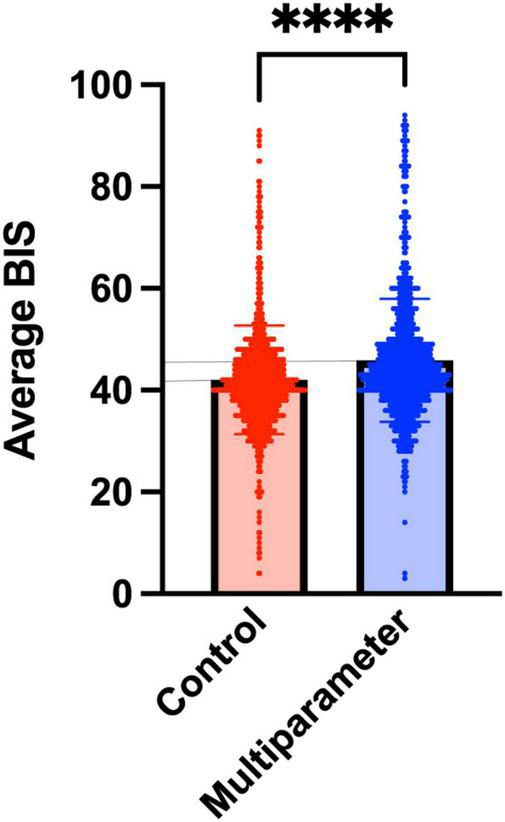
A comparison of average BIS value among the groups. Average BIS index values throughout the intervention. Red is the control group, and blue is the BIS multiparameter group. The bar represents the mean with their standard deviation. ****p < 0.0001. BIS, bispectral index.

Additionally, the patients managed with the new BIS multiparameter protocol had higher BIS values of 46.2 ± 12.2 compared to 41.9 ± 11 in the control group (*p* < 0.001). A daily comparison of BIS values between the two groups is available in Figure 2. The median SF95 and SR values in the intervention group are presented in Supplementary Figure 6 and Supplementary Table 7.

In both arms, there was a reduction in propofol use and an increase in midazolam use between days 1 and 5. The proportion of patients on propofol and/or midazolam during days 1–5 was not different for each group, as described in Supplementary Figure 9. Total adjusted midazolam administered during the first 5 days of the protocol was similar between the groups [median: 0.07 (IQR 0.05–0.09) mg/k/h in the control group vs. 0.06 (IQR 0.05–0.11) mg/k/h in the MP group, *p* = 0.005]. No differences were found on each day either (Supplementary Table 10). During the study, patients in the control group were on NMBs 4.3 [IQR 2.7–5] days compared to the MP group 4.4 [IQR 2.7–5] days (*p* = 0.90). A total of 34% of the enrolled patients had a suspected adverse event related to propofol infusion; hence, propofol was stopped and replaced by midazolam, similarly in the two groups. A detailed description of suspected AEs, specific causes, and possible PRIS cases in both groups is provided in (Supplementary Tables 11, 12). Additionally, we performed a sensitivity analysis with the subgroup of patients who completed this 5-day protocol with propofol (Supplementary Table 13).

In the *post hoc* analysis of the data, NMB use, daily dose of fentanyl, and prone position were associated with a higher daily dose of propofol (Supplementary Table 14). In the analysis of effect modification, the BIS multiparameter protocol reduced the dose of propofol by a daily mean of 963 mg (CI 95% –1,837 to –88 mg), and none of the studied variables (NMBs, fentanyl dose, prone position, and age) modified the effect of the intervention group (Supplementary Figure 15).

## Discussion

In the present study, we were not able to demonstrate the impact of a sedation guided by the BIS multiparameter protocol rather than the control group in increasing the 30-day VFD. However, we documented that the intervention led to a reduction in total propofol administration throughout the initial 5 days after randomization. Accordingly, patients in the intervention group exhibited higher mean BIS values during this period. Importantly, the effect of the intervention on the propofol dose was not modified by other factors associated with this outcome.

The reasons for the lack of difference in 30-day VFD despite achieving an almost 20% reduction in total propofol administration are as follows: The indication for deep sedation lasts longer than we previously expected (27, 28). Meaning that the intervention was only present for a fraction of the total time spent under deep sedation. Additionally, COVID-19 patients spent more time in MV compared to other ARDS groups, and therefore we overestimate our original 30-day VFD. Therefore, our sample size was insufficient to detect the expected difference, leaving the trial to underpower. Although we found a statistically significant difference between the groups in terms of the average BIS values through the days of the intervention, this difference does not seem to be clinically significant. Average BIS values were in the lower part of our desired range, meaning that despite the intervention protocol, there is still some room for improvement. This is also in agreement with the SEF95 and SR reported in the intervention group, whereas a proportion of the values were still beyond the desired target. These results may have several reasons: nurses may be concerned to have unexpected “awakening” episodes, especially in a scenario of high workload as in the pandemic; COVID-19 leads to a neuroinflammatory state (32) that may lead the brain to be more sensitive to hypnotic drugs (33). On the other hand, part of the effect may be lost because almost one-third of patients received midazolam by the end of the intervention period. This was mainly explained by concerns regarding potential PRIS, although we now know that severe COVID-19 has a noteworthy impact on lipid metabolism (34, 35). Finally, our intervention protocol does not help physicians to tailor opioid administration. Considering that fentanyl has also an unfavorable pharmacokinetic profile, it may also be playing a role in sedation-associated outcomes.

Nonetheless, our study had some important findings which may be relevant for clinical practice and subsequent studies. To our knowledge, this is the first clinical trial that suggests a significant reduction in propofol administration using an MP to guide deep sedation in patients with ARDS, besides the surgical setting (36). In theory, there may be several potential benefits of decreasing the propofol dose. First, there may be a decrease in the undesired effects of hypnotics in the ICU, such as hypotension, metabolic alterations, or PRIS. However, our results not shown significant differences in safety parameters studied. Second, we may avoid excessive propofol accumulation in body fat, diminishing the time needed to awaken a patient when deep sedation is no longer required (23). Third, reducing propofol administration may be useful to the drug shortage situation in the pandemic peak (3, 37). A reduction in propofol consumption is particularly useful in COVID-19 patients since it has been reported that patients with COVID-19 ARDS require higher doses of propofol and benzodiazepines than patients with non-COVID-19 ARDS (38).

It is important to remark that even with the reduced amount of propofol administered, we did not report any adverse event related to light sedation, such as unintended extubation. To avoid light sedation when the patient actually required deep sedation (most of the time accompanied by NMB), our intervention protocol started with a clinical goal of SAS 1–2 and a BIS target between 40 and 60. Therefore, we set a security boundary to avoid unintended light sedation, but at the same time, we aimed to avoid “to deep” sedation by avoiding both a lower SEF and a higher SR.

Deep sedation has been associated with worse outcomes in MV patients (5). When clinicians target deep sedation, burst suppression, which has been associated with delirium and mortality, frequently occurs (39). Since it has been recognized that even a short period of time is associated with adverse outcomes, we design out intervention to evaluate the SR every 2h and reduce the propofol infusion rate if this was over 2% (39). In a cohort of 11 patients with severe ARDS with COVID-19, the authors found that 58% of the frontal EEG evaluations performed by an epileptologist showed an excessive sedation pattern (40). Thus, improving brain monitoring with an intensified algorithm under these circumstances might minimize the occurrence of excessive sedation and its potential deleterious consequences. However, it is important to mention that some EEG patterns, such as burst suppression, may be a consequence of excessive sedative administration but also from brain damage (41, 42). The latter is of particular interest given the known brain effect of the SARS-CoV-2 (32).

In the ICU, the use of EEG-based monitors is controversial and the evidence is still scarce. However, recent consensus had recommended its use to monitor the level of sedation in all patients unsuitable for clinical evaluation (43). In neurocritical patients, a beneficial impact of BIS-guided sedation in addition to a clinical scale-guided protocol was reported (44). In ARDS patients, different groups have suggested the use of EEG monitoring (6), which is the reason that although recognizing that the use of a BIS monitor is not considered as “standard of care,” we decided to use a target of BIS 40–60 for our control group. Our results are the first suggesting benefits of adding other EEG-derived parameters to guided sedation in ARDS patients, at least in relation to the amount of propofol administered. We also showed that in a significant proportion of patients in which the BIS target was properly achieved, SEF95 and SR were still indicating an oversedation state (Supplementary Figure 16).

Processed EEG monitors such as BIS™ were originally developed to monitor anesthesia depth in the operative room. However, these processed EEG devices and their proprietary indexes have been largely discredited due to their lack of mechanistic underpinning and due to growing evidence that they are ineffective at monitoring unconsciousness and avoiding intraoperative awareness (45). In this regard, in the last decade, there has been a tremendous advance in the field, aiming to replace proprietary algorithms with more personalized and accurate metrics derived from the EEG signal. New generation devices now exhibit quantification of several other variables that are deeply related to the hypnotic drug modulation of neural oscillations. Some parameters may be obtained by analyzing the spectral features related to the signal, such as the SEF95, or may be obtained directly from the time series, such as the SR (46). Both a lower SEF95 (i.e., below 10 Hz) and a higher SR (over 2%) are signatures of a deep state of unconsciousness (i.e., oversedation) and are associated with poor outcomes (22, 39). Also, it is now recognized that even between the lower and upper edge for BIS index may have a clinical impact (47). Therefore, using a multiparameter approach may allow titrating drugs to maintain secure levels of sedation but avoid being in the inferior limit of the manufacturer’s recommendation target of BIS index. More important, our intervention aimed to guide deep sedation using parameters that are fundamental neurophysiological signatures of GABAergic hypnotic-induced unconsciousness (19).

Among the potential confounders between the intervention itself and the propofol administration dose, NMB agents are of particular interest in patients with COVID-19 and severe ARDS. Inoue et al. (48) reported that the NMB effect on the BIS depends on baseline BIS values, in agreement with the findings of Inoue et al., who reported a differential effect of NMBs on BIS values if patients were at moderate or deep sedation levels. It has also been reported that the sole NMBD administration may reduce BIS value (49). This is another argument to use other EEG-derived parameters to guide sedation in patients receiving neuromuscular block agents. NMBs use has been more frequently reported in COVID-19 patients, and that is concordant with our data. In the *post-hoc* analysis, we found that the daily use of NMB infusion was associated with higher daily propofol doses but that the NMBs did not modify the effect of the MP on propofol doses. The higher propofol dose in patients receiving NMBD may be a counterintuitive result. However, the underlying uncontrolled confounder beneath this observation may be the fact that the patients receiving NMBD are more seriously ill. What we observed and learned from COVID-19 patients is that when ARDS is severe, achieving deep sedation is challenging because the respiratory drive is notoriously high, with a higher incidence of ventilatory asynchrony and the associated challenge to achieve a protective ventilation. In this scenario, and to avoid further lung damage, physicians tried to further sedate the patients, and ultimately use NMBs.

The strengths of our study were the design, the pragmatic nature of the intervention, and the experience of our team in terms of the previous implementation of multimodal protocols (50). It is important to remark that our control group was already based on PADIS guidelines, including the use of BIS between 40 and 60 for deep sedation, which is already an exigent comparator group. We also consider that the protocol for intervention was easily adopted by our team of nurses.

Our study also has some limitations. First, it was a single-center study. External validation may also be limited, although our sedation protocol is based on PADIS guidelines. The sample size was calculated at the beginning of the SARS-CoV-2 pandemic when there was less experience with COVID-19 patients; therefore, we underestimated the time they would spend on MV. The VFD at day 30 found in our trial is not only inferior to what we expected in our sample size calculation but also regarding previous trials in ARDS (27, 28). Therefore, our trial might be underpowered. Likewise, our control group, which includes BIS monitoring, could underestimate the benefits of multimodal monitoring compared to only clinical scales. Plus, our intervention was designed to last 5 days; thus, a longer period with the intervention may be more effective in increasing the days without MV. However, this approach was considered unfeasible during the pandemic. Because of the trial design, treating nurses were not blinded to the group allocation, which may be a potential source of bias. Finally, the protocol was designed to be used in patients receiving GABAergic sedatives (such as propofol) and it won’t be applicable to patients receiving other types of sedatives such as ketamine or dexmedetomidine, since they exhibit different electroencephalographic signals (19).

Future studies may address these limitations and evaluate in a larger population if EEG-based protocol to guide sedation in critical care patients could be associated with favorable middle- and long-term outcomes.

## Conclusion

In conclusion, we demonstrated that an EEG-based protocol to guide deep sedation in severe COVID-19 patients is associated with a reduction in the total propofol administration 5 days after randomization, without modifying the VFD at day 30. These results highlight the importance of performing future multicenter trials aimed at evaluating multicomponent strategies to monitor deep sedation in ARDS patients.

## Data availability statement

The raw data supporting the conclusions of this article will be made available by the authors, without undue reservation.

## Ethics statement

The study protocol was reviewed and approved by the Ethics Committee from the Hospital Clinico de la Universidad de Chile. The legal representative of the patient provided their written informed consent to participate in this study.

## Author contributions

ET, AP, JE, and RG: conceptualization. JF and VR: methodology. ET, JF, AG, and RG: formal analysis and investigation. ET and RG: writing—original draft preparation. AP, DB, AG, and FM: writing—review and editing. RG, DB, and DP: funding acquisition. DP and VR: resources. DB and DP: supervision. All authors contributed to the article and approved the submitted version.
