# Supplementary material for: Electroencephalography spectral edge frequency and suppression rate-guided sedation in patients with COVID-19: A randomized controlled trial
Source: Front Med (Lausanne). 2022 Nov 4;9:1013430. doi: 10.3389/fmed.2022.1013430 (PMC9671936; doi:10.3389/fmed.2022.1013430)
Supplement: Supplementary file 1 [file Data_Sheet_1.docx]

Supplementary Material

**Table of contents**

| **Supplemental Method 1.** Institutional Analgesia and sedation protocol | Page 2 |
| --- | --- |
| **Supplemental Method 2.** Institutional Mechanical Ventilation and Weaning protocol | Page 3 |
| **Supplemental Figure 3.** Intervention flow-chart protocol for deep sedation | Page 4 |
| **Supplemental Method 4.** CONSORT Checklist | Page 5 |
| **Supplemental Method 5.** Protocol and Statistical Analysis Plan | Page 7 |
| **Supplemental Figure 6.** Median SEF95 and SR in the multiparameter group | Page 12 |
| **Supplemental Table 7.** Median SEF95 and SR in the multiparameter group per day of intervention | Page 13 |
| **Supplemental Figure 8.** Primary Outcome (30-days VFD) individual data distribution | Page 14 |
| **Supplemental Figure 9.** Propofol and midazolam administration rate through intervention | Page 15 |
| **Supplemental Table 10.** Midazolam administration rate and dose | Page 16 |
| **Supplemental Table 11.** Adverse events | Page 17 |
| **Supplemental Table 12.** Adverse events part 2 | Page 19 |
| **Supplemental Table 13.** Outcomes in patients who only receive propofol for sedation | Page 20 |
| **Supplemental Table 14.** Univariate analysis for confounders for daily propofol dose administered | Page 22 |
| **Supplemental Figure 15.** Effect modification of the MP-protocol on daily propofol dose. | Page 23 |
| **Supplemental Figure 16.** Association between BIS values and SEF95/SR. | Page 24 |
| **References** | Page 25 |

**Supplementary Material 1.** Institutional Analgesia and sedation protocol

**Evaluations:** They were carried out every 2 hours by nursing personnel trained with behavioural pain scale (BPS) for pain assessment, and sedation-agitation scale (SAS) to assess the level of sedation ^1, 2^. - Daily goal: it is defined daily by an intensivist staff, suggesting deep sedation (SAS 1-2) in case of PaO2/FiO2 <150, pH <7.25 and/or impossibility of complying with protective MV despite ventilatory management. In the case of PaO2/FiO2> 150, superficial sedation is prescribed with the patient in supine position.

**Drugs:** Our protocol contemplates the use of propofol as first line sedative in cases of a goal of deep sedation, and dexmedetomidine if a goal of light sedation. As adjunctive analgesia, we associated acetaminophen i.v. 1 g each 8 hours, and dipyrone 3-5 g per day if BPS> 5 persists.

**Start-up scheme:** It is recommended to start with fentanyl 0.6 ug/kg/h + paracetamol 1 gr iv every 8 hours + dexmedetomidine 0.8 ug/kg/h in case of SAS 3-4 goal, and with fentanyl 1.2 ug/kg/h + paracetamol 1 gr iv every 8 hours, plus propofol 1.5 mg/kg/h if target SAS 1-2. A brief description, Figure, and dose adjustment in Supplementary Material 2 (SM2).

In patients using propofol, daily measurement of creatinine kinase (CK), triglycerides and lactate were suggested for early evaluation of the risk of propofol-related infusion syndrome (PRIS). The use of midazolam infusion is suggested in cases where it is necessary to stop propofol, or in addition to it when the maximum propofol dose (3 mg/kg/h) is reached without achieving the sedation goals.

**Neuromuscular block agents administration:** It was suggested to use if PaO2/FiO2 <120 and/or impossibility of complying with protective MV (Driving pressure > 15 cm H20). The first line drug is cisatracurium, but in the absence of this in the national market, it was defined to use rocuronium or atracurium in infusion guided to Train of Four 0-1.

**
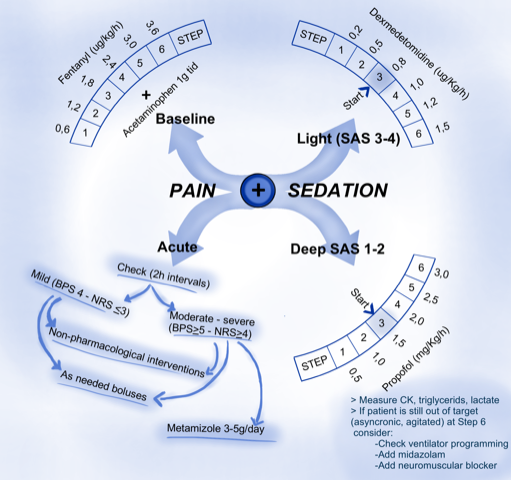
**

**Supplementary Material 2.** Institutional Mechanical Ventilation protocol and Weaning

COVID-19 Management Institutional Protocol

From July 2020, the management included daily dexamethasone 6 mg i.v. for ten days, an early trial of high-flow nasal cannulation (HFNC) if respiratory insufficiency and signs of increased work of breathing were present, and awake prone positioning if tolerated. Patients were started on MV if they needed urgent intubation in the emergency room or had HFNC failure. All patients followed an institutional protocol for MV that included standardized use of analgesia and sedation, protective MV, MV weaning and tracheostomy if needed.

Mechanical Ventilation protocol

At the early phase of MV, all the patients were ventilated under A/C volume-controlled mode using tidal volume (V_T_) of 6 ml/kg of predicted body weight. The best compliance during the decremental PEEP trial was used to select the PEEP level ^3^. Respiratory Rate was adjusted to a pH > 7.35. To ensure protective MV, Plateau Pressure and Driving pressure (DP) were maintained lower than 30 and 15 cmH2O, respectively. If the patient presented DP >15 cmH2O while receiving V_T_ 6 mL/kg PBW, we decreased VT until achieving DP < 15 (active humidification instead of heat and moisture exchanger filter was preferred in these cases). Prone position was indicated if the patient presented persistent PaO2:FiO2 less than 150 mmHg ^4^. In our centre, a protocol of prolonged prone position for severe ARDS patients, defined 15 years ago and actualized for this pandemic period, was performed ^5^. Extracorporeal Life Support Organization (ELSO) COVID-19 guidelines were implemented for the most severe ARDS cases, including patient selection criteria and ECMO management ^6^.

Institutional Weaning and tracheostomy protocol

All the patients were daily evaluated for the presence of criteria for spontaneous breathing trial (SBT). If the patient has it, it was done in PS 5, PEEP 5 cm H20 for 30-60 min. If the patient has installed a tracheostomy, we have a local protocol for intermittent disconnection. A percutaneous tracheostomy was done bedside by intensivist if MV duration > 10-14 days and the global status suggests that the patient has intermediate to high possibilities to discharge alive.

**Supplemental Figure 3.** Intervention flow-chart protocol for deep sedation


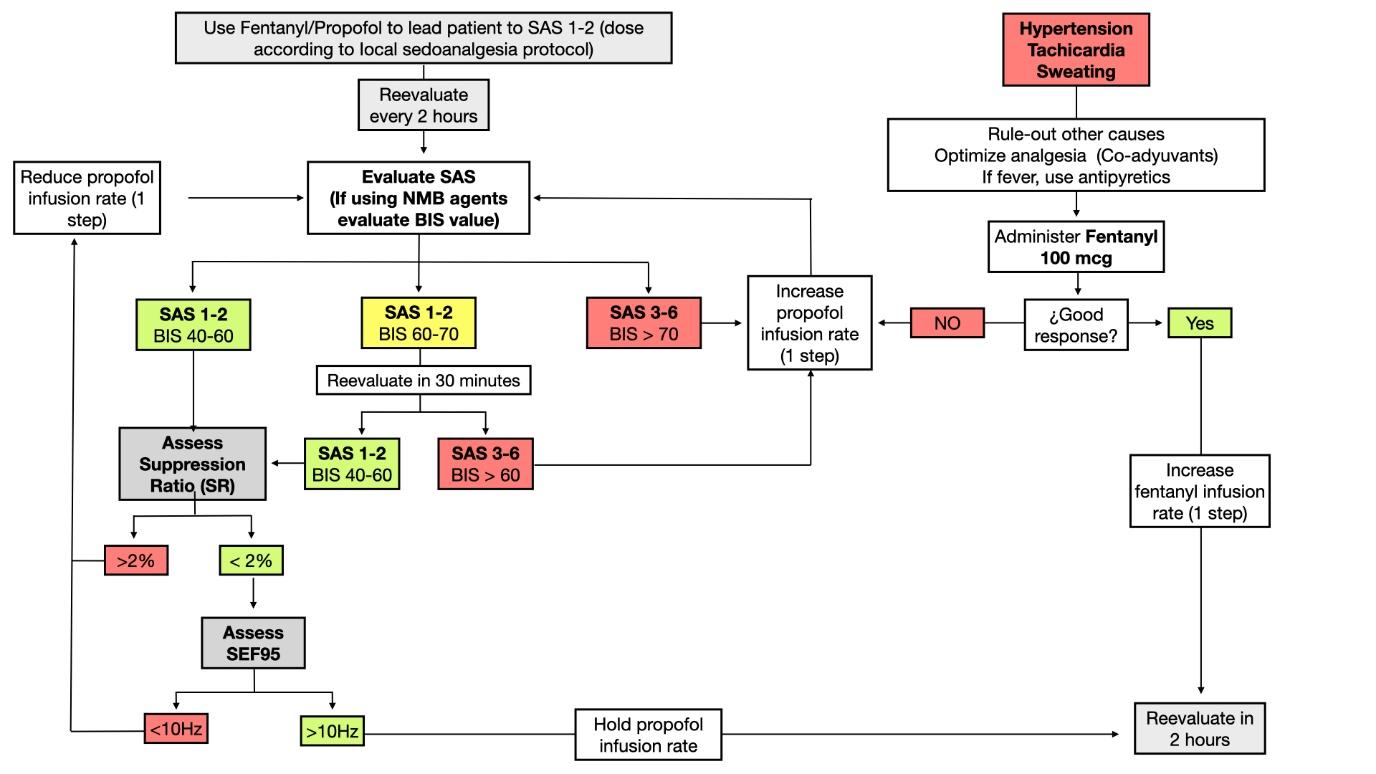


**Supplementary Figure 3.** Intervention flow-chart protocol for deep sedation. *SAS = Sedation-agitation scale; BIS = Bispectral Index; 1 step reduction means a 0.5 mg/k/h reduction in the infusion rate.*

**Supplementary Material 4.** CONSORT Checklist

/CONSORT 2010 checklist of information to include when reporting a randomised trial*

| Section/Topic | Item No | Checklist item | Reported on page No |
| --- | --- | --- | --- |
| Title and abstract | | | |
|  | 1a | Identification as a randomised trial in the title | 1 |
|  | 1b | Structured summary of trial design, methods, results, and conclusions (for specific guidance see CONSORT for abstracts) | 3-4 |
| Introduction | | | |
| Background and objectives | 2a | Scientific background and explanation of rationale | 5-7 |
|  | 2b | Specific objectives or hypotheses | 5-7 |
| Methods | | | |
| Trial design | 3a | Description of trial design (such as parallel, factorial) including allocation ratio | 8 |
|  | 3b | Important changes to methods after trial commencement (such as eligibility criteria), with reasons | NA |
| Participants | 4a | Eligibility criteria for participants | 8 |
|  | 4b | Settings and locations where the data were collected | 8 |
| Interventions | 5 | The interventions for each group with sufficient details to allow replication, including how and when they were actually administered | 9 |
| Outcomes | 6a | Completely defined pre-specified primary and secondary outcome measures, including how and when they were assessed | 10-11 |
|  | 6b | Any changes to trial outcomes after the trial commenced, with reasons | NA |
| Sample size | 7a | How sample size was determined | 11 |
|  | 7b | When applicable, explanation of any interim analyses and stopping guidelines | NA |
| Randomisation: |  |  |  |
| Sequence generation | 8a | Method used to generate the random allocation sequence | 8 |
|  | 8b | Type of randomisation; details of any restriction (such as blocking and block size) | 8 |
| Allocation concealment mechanism | 9 | Mechanism used to implement the random allocation sequence (such as sequentially numbered containers), describing any steps taken to conceal the sequence until interventions were assigned | 8 |
| Implementation | 10 | Who generated the random allocation sequence, who enrolled participants, and who assigned participants to interventions | 8 |
| Blinding | 11a | If done, who was blinded after assignment to interventions (for example, participants, care providers, those assessing outcomes) and how | 8 |
|  | 11b | If relevant, description of the similarity of interventions | 9 / SM1-2 |
| Statistical methods | 12a | Statistical methods used to compare groups for primary and secondary outcomes | 11-12 |
|  | 12b | Methods for additional analyses, such as subgroup analyses and adjusted analyses | 11-12 |
| Results | | | |
| Participant flow (a diagram is strongly recommended) | 13a | For each group, the numbers of participants who were randomly assigned, received intended treatment, and were analysed for the primary outcome | 13 – Fig1 |
|  | 13b | For each group, losses and exclusions after randomisation, together with reasons | 13- Fig1 |
| Recruitment | 14a | Dates defining the periods of recruitment and follow-up | 13 |
|  | 14b | Why the trial ended or was stopped | NA |
| Baseline data | 15 | A table showing baseline demographic and clinical characteristics for each group | Table 1 - 13 |
| Numbers analysed | 16 | For each group, number of participants (denominator) included in each analysis and whether the analysis was by original assigned groups | Table 1 and 2  13-14 |
| Outcomes and estimation | 17a | For each primary and secondary outcome, results for each group, and the estimated effect size and its precision (such as 95% confidence interval) | 13-14 |
|  | 17b | For binary outcomes, presentation of both absolute and relative effect sizes is recommended | 13-14 |
| Ancillary analyses | 18 | Results of any other analyses performed, including subgroup analyses and adjusted analyses, distinguishing pre-specified from exploratory | 13-14 |
| Harms | 19 | All important harms or unintended effects in each group (for specific guidance see CONSORT for harms) | SM 8-10 |
| Discussion | | | |
| Limitations | 20 | Trial limitations, addressing sources of potential bias, imprecision, and, if relevant, multiplicity of analyses | 19 |
| Generalisability | 21 | Generalisability (external validity, applicability) of the trial findings | 18-19 |
| Interpretation | 22 | Interpretation consistent with results, balancing benefits and harms, and considering other relevant evidence | 15-18 |
| Other information | | |  |
| Registration | 23 | Registration number and name of trial registry | 8 |
| Protocol | 24 | Where the full trial protocol can be accessed, if available | NA |
| Funding | 25 | Sources of funding and other support (such as supply of drugs), role of funders | 2 |

**Supplementary Material 5.** Protocol and Statistical Analysis Plan

SAP Version: V3 2021-12-05

Investigators:

Eduardo Tobar, MD.

Ignacio Farías.

Rodrigo Gutiérrez, MD, PhD.

Statistical Advisor

Abraham Gajardo, MD, PhD.

**Amendment History**

| **Version** | **Date** | **Item / Section** | **Details** |
| --- | --- | --- | --- |
| V1 | 2020-12-20 | SAP/all sections | Initial SAP V1 |
| V2 | 2021-10-14 | Sensitivity analysis | Include a sensitivity analysis for those patients that only received propofol for sedation in the first 5 days |
| V3 | 2021-10-28 | Secondary outcomes | We modified some definitions regarding the following secondary outcomes: ICU length of stay, Hospital length of stay. We add mortality at 90 days and tracheostomy rate. Accidental extubation was removed from secondary outcomes and considered as adverse event. |
| V4 | 2021-11-25 | Secondary outcomes | We eliminated a secondary outcome due to technical issues regarding plasmatic propofol concentration determination with HPLC |
| V5 | 2021-12-05 | Post-hoc analysis | Post-hoc mixed effect model to explore the association between some patient’s factors and propofol doses |

**Background**

Deep sedation in patients with COVID-19 may be challenging in many aspects. The use of an BIS multiparameter protocol to guide deep sedation may be useful in this population, considering their unusually high sedation requirements. In the present trial, we aim to evaluate an EEG-based protocol to guide deep sedation in patients with COVID19, using to EEG derived parameters that are displayed in the BIS monitor: Suppression Rate and Spectral Edge Frequency. The protocol is designed to both minimize the suppression rate along with maintaining a spectral edge frequency over 10 Hz. The use of this protocol may reduce the amount of sedatives administered and, therefore, diminish the time needed for the weaning process.

**Study Design**

**Patient eligibility criteria**

| **Table: Inclusion and Exclusion Criteria** | |
| --- | --- |
| **Inclusion Criteria** | **Exclusion Criteria** |
| - Male and female patients 18 years old and older. - Admitted to the ICU due to severe COVID-19 pneumonia requiring MV | - Patients with contraindication to receive propofol or fentanyl. - Known history of chronic liver disease Child C stage - Known history of end-stage kidney chronic disease |

**Hypothesis:** Deep sedation guided by a protocol based on intensive use of BIS monitoring parameters (BIS, SEF95 and SR) will increase ventilator-free days (VFD) at 30 days compared with a control group in mechanically ventilated COVID-19 patients.

Design: Randomized parallel control trial

Groups:

- Control
- Intervention:

Location: Hospital Clínico de la Universidad de Chile, Santiago, Chile.

**Outcomes**

**Primary**

| **Name** | **Definition** | **Unit** | **Time Frame** | **Type** |
| --- | --- | --- | --- | --- |
| Ventilator-free days at 30 days | Number of days in which the patient is both alive and out of the invasive mechanical ventilator | Days | Day 30 after randomization | Numeric |

**Secondary**

| **Name** | **Definition** | **Unit** | **Time Window** | **Type** |
| --- | --- | --- | --- | --- |
| Accumulate propofol administered | Total propofol dose administered to a patient through the window time | mg | Day 5 after randomization | Numeric |
| Total adjusted propofol administered | Total propofol dose administered to a patient through the window time, adjusted by patient weight (in kg) and by the duration of the infusion (h) | mg/k/h | Day 5 after randomization | Numeric |
| Total adjusted fentanyl administered | Total fentanyl dose administered to a patient through the window time, adjusted by patient weight (in kg) and by the duration of the infusion (h) | mcg/k/h | Day 5 after randomization | Numeric |
| PRIS | For the PRIS definition, we used the one suggested by Roberts et al.^7^. | Number of patients | Day 30 after randomization | Binary |
| Delirium incidence | Delirium incidence was defined as the presence of any positive CAM-ICU test during MV | Number of patients | Day 30 after randomization | Binary |
| Tracheostomy rate | Number of patients with a percutaneous or surgical tracheostomy | Number of patients | Day 30 after randomization | Binary |
| Success of the first ventilator weaning trial | success of the first weaning trial was defined as suggested by the WIND trial^8,9^. | Number of patients | Day 30 after randomization | Binary |
| Days alive and out of hospital | Number of days in which the patient was alive and discharged from the hospital | Days | Day 90 after randomization | Numeric |
| 30-day mortality | Confirmed deceased patient | Number of patients | Day 30 after randomization | Binary |
| 90-day mortality | Confirmed deceased patient | Number of patients | Day 90 after randomization | Binary |

**Sensitivity Analysis**

Primary and secondary outcomes will be explored independently in the next subgroups:

1. Patients that only received propofol.
2. Obese patients
3. NMB
4. Age

**Post-hoc Analysis**

**Linear Mixed Effect Model:** According to previous research, patient age, sex, fentanyl use, use of prone positioning, and neuromuscular blockade have been associated with the dose of propofol^10,11^. Indeed, we decided to explore the association between these factors and propofol doses through mixed effects linear regression. Then, we explored the effect modification of each variable in linear regression models including the intervention, the interaction of variables and the interaction between them as fixed effects. In the case of age, we used a cutoff value of 65 years. In the case of prone positioning and NMBs, because both vary by day during the follow-up, we considered daily data.

**Other exploratory analysis**

As exploratory analysis we compared:

1. Coma days: to explore the number of days in which the patient was deeply sedated.
2. Delirium days: to explore the duration of the delirium episodes
3. Propofol suspension: to explore the rate of propofol suspension in each group, at its reasons.
4. Peak norepinephrine infusion rate: to explore the maximal infusion rate of norepinephrine in each group.

**Adverse events**

We evaluate the following adverse events:

1. Unprogrammed extubation
2. Hemodynamic variables
   1. Heart Rate
   2. Systolic Blood Pressure
   3. Mean Blood Pressure

**Sample size calculation**

No reliable data were available at the trial design to allow for an accurate sample size calculation, regarding the potential benefit of intensified BIS monitoring in VFD in patients with ARDS. Therefore, we used data from other studies evaluating different interventions to increase VFD. Previous studies have documented an 20% increase in VFD at 30 days with PEEP trials interventions^42,43^. We estimated that the BIS multiparameter group would have 18$\pm$3 VFD at 30 days compared to 15$\pm$3 in the control group^.^ To detect this 20% effect size with a power of 80%, a two-tailed alpha of 0.05, and a 10% drop-out rate, we estimated 25 patients per group.

**Statistical Analysis**

Categorical variables are presented as numbers (percentages) and were compared by Fisher’s exact test. Continuous variables are presented as the mean (standard deviation [SD]) or the median (interquartile range [IQR] depending on the data distribution evaluated by the Shapiro–Wilk test; two-group comparisons were made by Student’s t test or Mann–Whitney’s U test, respectively.

An intention-to-treat analysis was performed for the primary outcome. We additionally analyzed the primary outcome only in those patients who received propofol for deep sedation, and we detailed the reported causes for propofol suspension. For secondary outcomes, the level of significance was adjusted with Bonferroni correction. Finally, a post hoc exploratory analysis was performed to assess the independent effect of the BIS multiparameter protocol on the use of sedatives adjusting with known potential confounders. For each variable, we performed mixed effects linear regression, considering the daily accumulated propofol dose as a dependent variable, each patient as a random effect, and the fixed effects (age, sex, fentanyl use, use of prone positioning, and neuromuscular blockade). All analyses were performed in Stata v.14 (Texas, USA) and Prism GraphPad v9.2 (California, USA).

**Supplementary Figure 6.** Median SEF95 and SR in the multiparameter group.

**
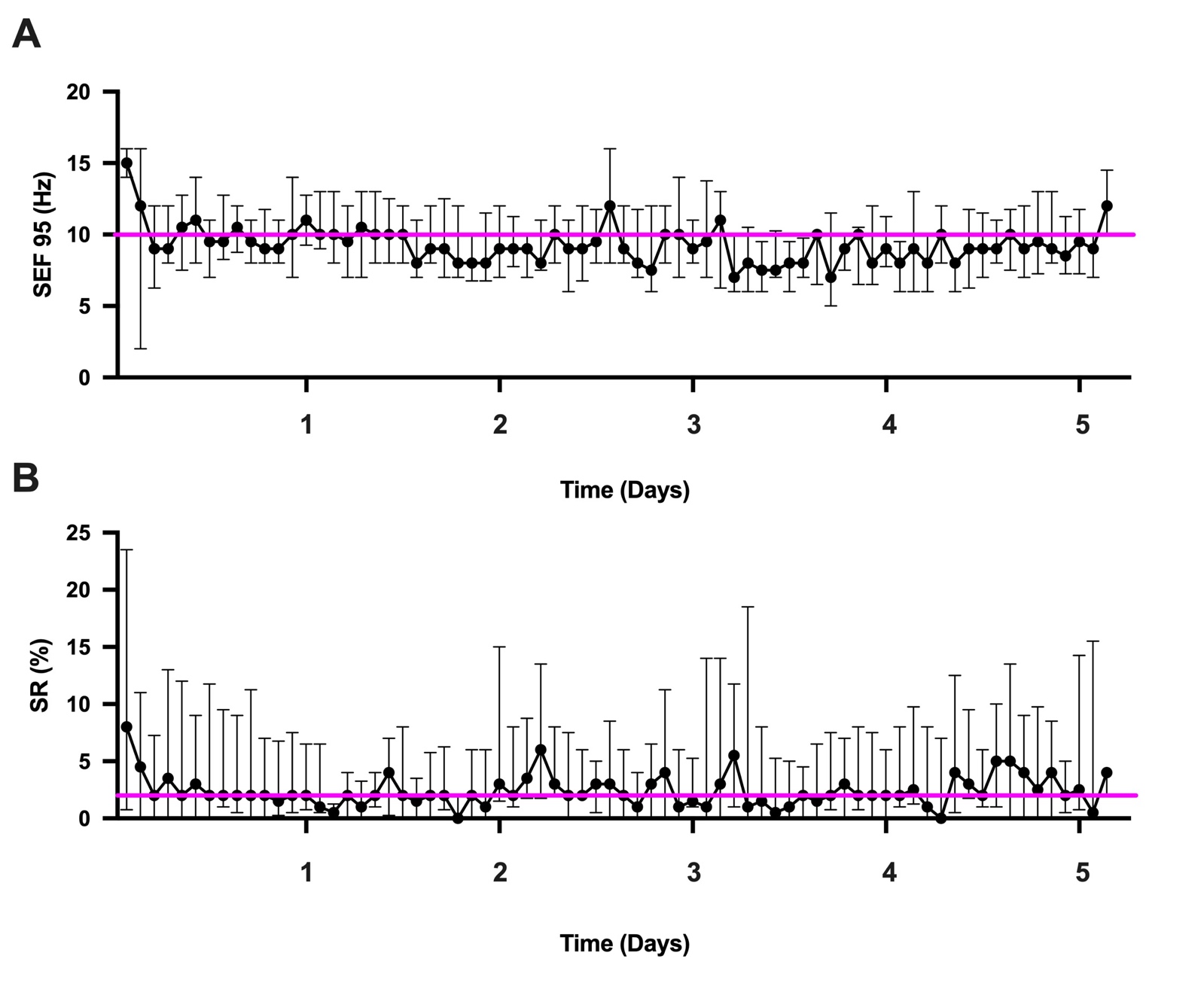
**

The median and the IQR for the SEF95 (A) and SR (B) in the multiparameter group during the intervention period are presented. Data was obtained from the clinical records. Cian horizontal line represents the corresponding target value for each parameter according to the intervention protocol.

**Supplementary Table 7.** Median SEF95 and SR in the multiparameter group per day of intervention.

| **Variable** | **Day 0**  **(n=25)** | **Day 1**  **(n=25)** | **Day 2**  **(n=19)** | **Day 3**  **(n=17)** | **Day 4**  **(n=11)** |
| --- | --- | --- | --- | --- | --- |
| SEF95 (Hz) | 10 (7.5-12) | 9 (8-12) | 10 (8-12) | 8 (7-11) | 9 (7-11.5) |
| SR (%) | 1.8 (0-9.8) | 1 (0-4) | 2.5 (1.8-5.3) | 2 (0-7.8) | 2.5 (1-6) |

*SEF95: Spectral Edge Frequency 95*

*SR: Suppression Rate*

**Supplementary Figure 8.** Primary Outcome (30-days VFD) individual data distribution

| **Control** | **Multiparameter** |
| --- | --- |
| 0 | 0 |
| 0 | 0 |
| 20 | 0 |
| 0 | 0 |
| 13 | 0 |
| 0 | 21 |
| 20 | 16 |
| 11 | 0 |
| 13 | 0 |
| 0 | 21 |
| 21 | 0 |
| 0 | 22 |
| 17 | 0 |
| 0 | 19 |
| 0 | 21 |
| 25 | 15 |
| 11 | 0 |
| 0 | 24 |
| 0 | 16 |
| 19 | 24 |
| 0 | 24 |
| 0 | 0 |
| 21 | 0 |
| 21 | 23 |
| 24 | 0 |


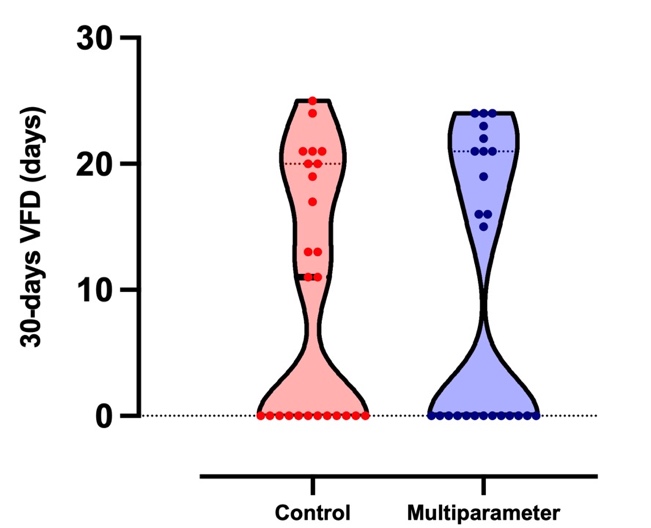


Violin plot presenting the median and interquartile range.

**Supplementary Figure 9.** Propofol and midazolam administration rate through intervention


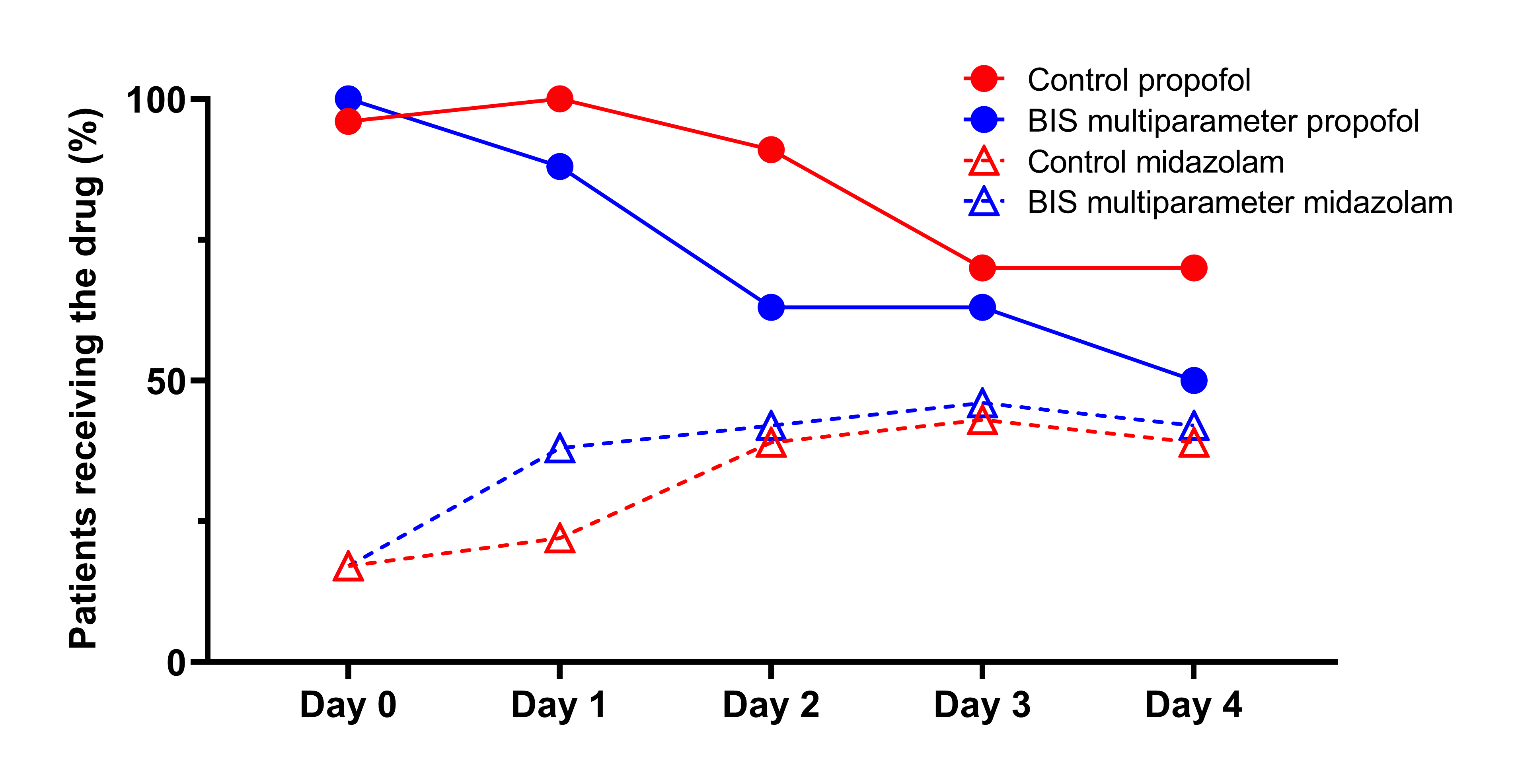


**Supplementary Table 10. Midazolam administration rate and dose**

|  | **Control (n=25)** | **Multiparameter protocol (n=25)** | **p-value** |
| --- | --- | --- | --- |
| Number of patients receiving midazolam |  |  |  |
| Day 1 |  |  |  |
| Day 2 |  |  |  |
| Day 3 |  |  |  |
| Day 4 |  |  |  |
| Day 5 |  |  |  |
| Accumulated adjusted dose (mg/k/h) |  |  |  |
| Day 1 | 0.09 (0.09 - 0.1) | 0.06 (0.03-0.1) | 0.21 |
| Day 2 | 0.06 (0.05 – 0.09) | 0.06 (0.03-0.06) | 0.24 |
| Day 3 | 0.05 (0.04 – 0.08) | 0.06 (0.05-0.09) | 0.34 |
| Day 4 | 0.06 (0.06 – 0.08) | 0.09 (0.06-0.17) | 0.59 |
| Day 5 | 0.08 (0.06 – 0.1) | 0.07 (0.04-0.15) | 0.59 |

**Supplementary Table 11.** Adverse events

|  | **Control (n=25)** | **Multiparameter protocol (n=25)** | **p-value** |
| --- | --- | --- | --- |
| **Propofol discontinuation***^‡^* |  |  |  |
| Number of patients, number (%) | 7 (28) | 9 (36) | 0.76 |
| Suspension day – days, median (IQR) | 2 (1 – 3) | 2 (2 – 3) | 0.69 |
| **Causes of suspension***^‡^* |  |  |  |
| Increased triglycerides, number (%) | 3 (12) | 1 (4) | 0.5 |
| Triglycerides level at the time of suspension – mg/dL, median (IQR) | 451 (440 – 510) | 675 (675 – 675) | 0.18 |
| Increased creatine kinase, number (%) | 4 (16) | 7 (28) | 0.61 |
| Creatine kinase level at the time of suspension – u/L, median (IQR) | 2,324 (2.187 – 3.058) | 1,059 (736 – 3.752) | 0.26 |
| Suspension for unknown reason, number (%) | 0 (0) | 1 (4) | 0.99 |
| PRIS incidence^*^ | 0 (0) | 0 (0) | 0.99 |
| **Suspect PRIS clinical manifestations***^‡ *^* |  |  |  |
| Cardiac dysfunction, number (%) | 0 (0) | 0 (0) | 0.99 |
| Metabolic acidosis, number (%) | 1 (4) | 0 (0) | 0.99 |
| Rhabdomyolysis, number (%) | 0 (0) | 0 (0) | 0.99 |
| Hypertriglyceridemia, number (%) | 4 (16) | 3 (12) | 0.99 |
| Renal failure, number (%) | 1 (4) | 1 (4) | 0.99 |
| Hepatic transaminitis, number (%) | 1 (4) | 3 (12) | 0.61 |
| Delirium days - days, median (IQR)^a^ | 0 (0-1) | 1 (0-3) | 0.25 |
| Coma days – days, median (IQR)^a^ | 14 (8-27) | 9 (8-22) | 0.28 |
| Unprogrammed extubation – yes, No. (%)*^‡^* | 0 (0) | 0 (0) | 0.95 |
| Peak norepinephrine infusion rate – mcg/k/min, median (IQR) *^‡^* | 0.05 (0.02-01) | 0.06 (0.02-0.08) | 0.99 |

*^‡^During the first 5 days after randomization.*

*^*^PRIS criteria described by Robert et al. (2009), associated with propofol infusion.*

^a^At day 30

**Supplementary Table 12.** Adverse events part 2

Hemodynamic parameters during sedation

|  | **Control (n=25)** | **Multiparameter protocol (n=25)** | **p-value** |
| --- | --- | --- | --- |
| **Heart rate** |  |  |  |
| Day – 1 – beats per minute, median (IQR) | 68 (59 – 84) | 65 (53 – 80) | 0.02 |
| Day – 2 – beats per minute, median (IQR) | 70 (60 – 86) | 69 (59 – 82) | 0.4 |
| Day – 3 – beats per minute, median (IQR) | 68 (60 – 84) | 72 (60 – 87) | 0.23 |
| Day – 4 – beats per minute, median (IQR) | 74 (64 – 92) | 71 (56 – 85) | 0.005 |
| Day – 5 – beats per minute, median (IQR) | 73 (65 – 91) | 76 (59 – 90) | 0.16 |
| **Systolic pressure** |  |  |  |
| Day – 1 – mmHg, median (IQR) | 116 (106 – 127) | 114 (105 – 128) | 0.36 |
| Day – 2 – mmHg, median (IQR) | 120 (110 – 131) | 117 (116 – 128) | 0.06 |
| Day – 3 – mmHg, median (IQR) | 120 (110 – 132) | 121 (109 – 136) | 0.48 |
| Day – 4 – mmHg, median (IQR) | 123 (113 – 136) | 122 (112 – 135) | 0.7 |
| Day – 5 – mmHg, median (IQR) | 124 (110 – 139) | 124 (113 – 143) | 0.36 |
| **Mean arterial pressure** |  |  |  |
| Day – 1 – mmHg, median (IQR) | 81 (75 – 87) | 79 (73 – 87) | 0.03 |
| Day – 2 – mmHg, median (IQR) | 84 (77 – 90) | 81 (75 – 90) | 0.06 |
| Day – 3 – mmHg, median (IQR) | 83 (77 – 90) | 84 (77 – 83) | 0.66 |
| Day – 4 – mmHg, median (IQR) | 83 (76 – 92) | 84 (76 – 93) | 0.66 |
| Day – 5 – mmHg, median (IQR) | 83 (76 – 92) | 86 (78 – 94) | 0.17 |

*^IQR: Interquartile Range^*

**Supplementary Table 13.** Outcomes in patients who only receive propofol for sedation

|  | **Control (n=14)** | **Multiparameter protocol (n=13)** | **p-value** |
| --- | --- | --- | --- |
| **Propofol use** |  |  |  |
| Day – 1, number (%) | 14 (100) | 13 (100) | 0.99 |
| Day – 2, number (%) | 14 (100) | 13 (100) | 0.99 |
| Day – 3, number (%) | 13 (93) | 10 (77) | 0.33 |
| Day – 4, number (%) | 13 (93) | 10 (77) | 0.33 |
| Day – 5, number (%) | 11 (79) | 9 (69) | 0.68 |
| **Propofol consumption*^†^*** |  |  |  |
| Total propofol consumption – mg, median (IQR) | 21,240 (17,830 – 25,500) | 15,560 (11,560 – 16,720) | 0.03 |
| Propofol infusion rate – mg/k/h, median (IQR) | 2.3 (1.9 – 2.5) | 1.9 (1.6 – 2.2) | 0.04 |
| ***ICU baseline physiological parameters^†^*** |  |  |  |
| NMB – yes, number (%) | 13 (93) | 13 (100) | 0.99 |
| APACHE II – score, median (IQR) | 16 (14 – 20) | 16 (14 – 17) | 0.54 |
| ***Primary Outcome*** |  |  |  |
| Ventilator free days– days, median (IQR)^†^ | 12 (0 – 21) | 15 (0 – 21) | 0.96 |
| ***Secondary Outcomes*** |  |  |  |
| Total fentanyl consumption – mcg/kg/h, median (IQR)^‡^ | 2.4 (1.7 – 3.0) | 2.5 (2.1 – 3.1) | 0.14 |
| Peak norepinephrine infusion – mcg/kg/min, median (IQR) ^‡^ | 0.03 (0.02 – 0.06) | 0.05 (0.02 – 0.08) | 0.45 |
| Delirium incidence*^†^* | 5 (36) | 6 (46) | 0.70 |
| Successful Weaning*^†^* | 7 (50) | 7 (54) | 0.99 |
| Hospital and alive free days, days, median (IQR)^£^ | 36 (3 – 49) | 52 (6 – 66) | 0.58 |
| 30-day Mortality – yes, number (%) | 1 (7) | 0 (0) | 0.99 |
| 90-day Mortality – yes, number (%) | 2 (14) | 0 (0) | 0.48 |

*^†^At day 30.; ^£^ At day 90.; ^‡^During the first 5 days after randomization.*

**Supplementary Table 14.** Univariate analysis for confounders for daily propofol dose administered

Effect of predefined variables on daily propofol dose

| **Variable** | **Mean effect (CI 95%) **** | **p-value** |
| --- | --- | --- |
| Neuromuscular blockade use | 960 (59 – 1862) | 0.037 |
| Daily fentanyl dose * | 30 (16 – 44) | <0.001 |
| Daily Midazolam dose * | -108 (-661 – 446) | 0.703 |
| Prone positioning | 685 (79 – 1291) | 0.027 |
| Elderly | -418,425 (-1397 – 426) | 0.402 |
| Male sex | 831,982 (-84 – 1748) | 0.075 |

*^*^ For each 100 units*

*^**^ Mean effect on daily propofol dose (mg) estimated by crude beta coefficient in mixed linear regression models*

**Supplemental Figure 15.** Effect modification of the MP-protocol on daily propofol dose.


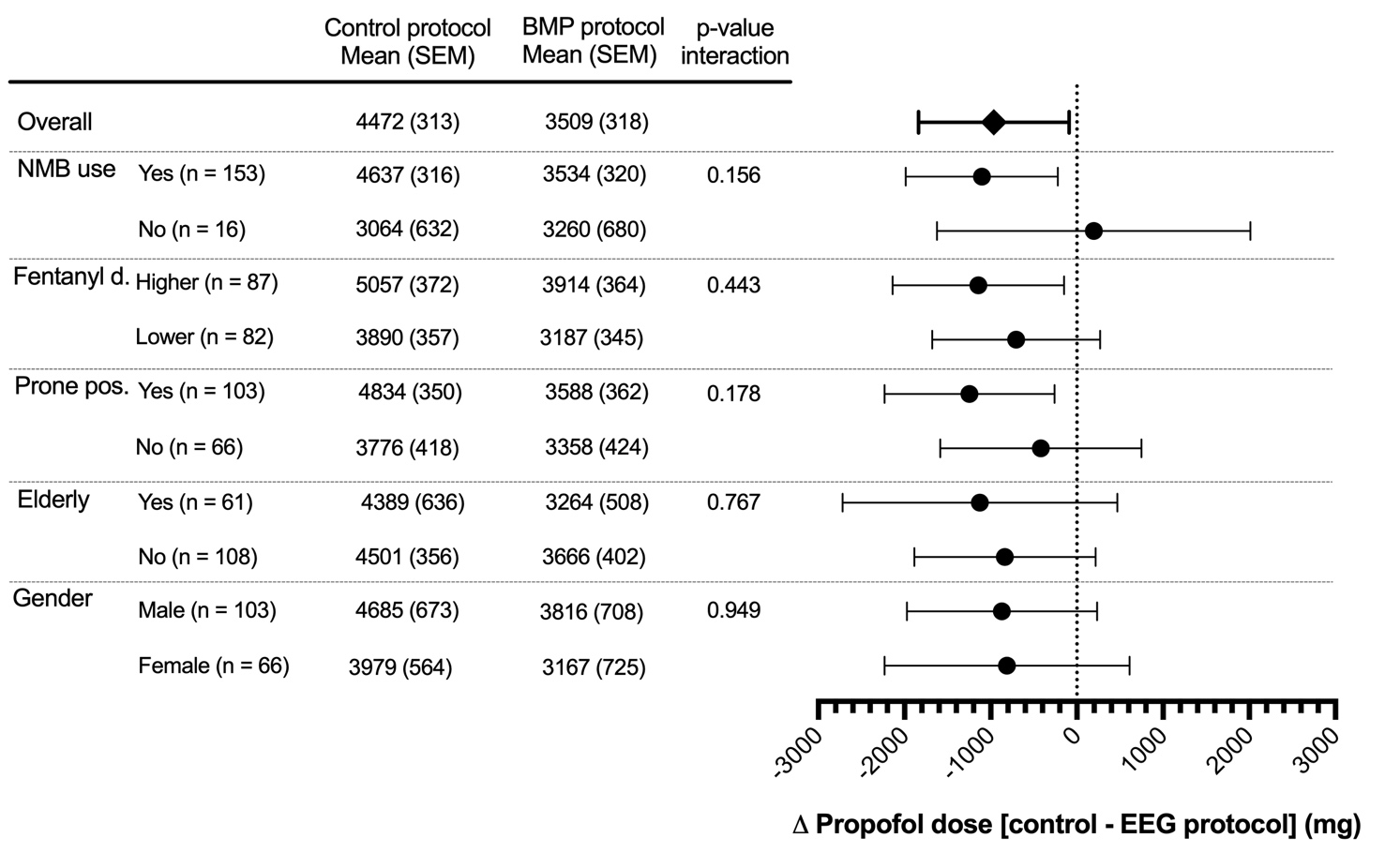


**Supplemental Figure 15.** **Effect modification of the MP-protocol on daily propofol dose**. The mean effect on daily propofol dose is shown at overall and by predefined sub-groups (left side). Also, the mean difference (and the respective 95% confidence interval) between MP-protocol and control groups are plotted (right side). *SEM: Standard error of mean; NMB: Neuro-muscular blockage, d.: dose, pos.: positioning*.

**Supplemental Figure 16.** Association between BIS values and SEF95/SR.

**
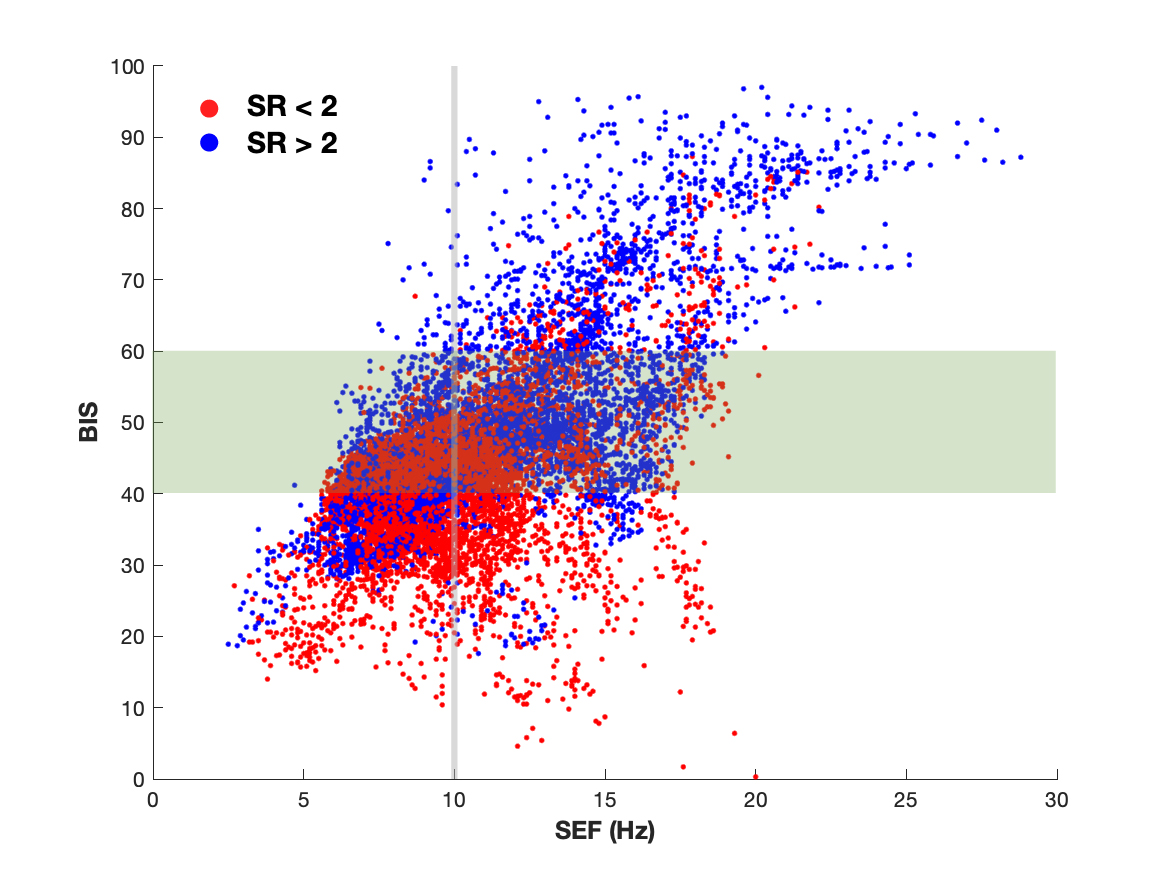
**

Data extracted directly from BIS Vista monitor was analyzed. Green area represents the target values for BIS, while the vertical grey line represent the SEF threshold established in the protocol. Datapoints in which SR was over 2% are presented in red. We found that among the 17501 data points in which BIS values were between the target range, 3178 presented a SEF95 value below 10 (18.8%) and 3291 a SR higher than 2% (18.8%). Because of restrictions in the hospital during pandemic, this data was obtained after the recruitment period when monitors were removed from the COVID ICU. As a consequence, metadata for these raw values directly extracted from the monitors is not trackable and therefore, this result should be interpreted cautiously.

**References**

1. Payen J-F, Bru O, Bosson J-L, et al. Assessing pain in critically ill sedated patients by using a behavioral pain scale. Critical Care Medicine 2001; **29**: 2258–63

2. Riker RR, Picard JT, Fraser GL. Prospective evaluation of the Sedation-Agitation Scale for adult critically ill patients. Critical Care Medicine 1999; **27**: 1325–9

3. Cornejo RA, Díaz JC, Tobar EA, et al. Effects of Prone Positioning on Lung Protection in Patients with Acute Respiratory Distress Syndrome. American Journal of Respiratory and Critical Care Medicine 2013; **188**: 440–8

4. Guérin C, Reignier J, Richard J-C, et al. Prone Positioning in Severe Acute Respiratory Distress Syndrome. New England Journal of Medicine 2013; **368**: 2159–68

5. Romero CM, Cornejo RA, Gálvez LR, et al. Extended prone position ventilation in severe acute respiratory distress syndrome: A pilot feasibility study. Journal of Critical Care 2009; **24**: 81–8

6. Diaz RA, Graf J, Zambrano JM, et al. Extracorporeal Membrane Oxygenation for COVID-19–associated Severe Acute Respiratory Distress Syndrome in Chile: A Nationwide Incidence and Cohort Study. American Journal of Respiratory and Critical Care Medicine 2021; **204**: 34–43

7. Roberts RJ, Barletta JF, Fong JJ, et al. Incidence of propofol-related infusion syndrome in critically ill adults: a prospective, multicenter study. *Critical Care*. 2009;13(5). doi:10.1186/cc8145

8. Tobar E, Romero C, Galleguillos T, et al. Confusion assessment method for diagnosing delirium in ICU patients (CAM-ICU): Cultural adaptation and validation of the Spanish version | M?todo para la evaluaci?n de la confusi?n en la unidad de cuidados intensivos para el diagn?stico de del?rium: adapt. *Medicina Intensiva*. 2010;34(1). doi:10.1016/j.medin.2009.04.003

9. Béduneau G, Pham T, Schortgen F, et al. Epidemiology of Weaning Outcome according to a New Definition. The WIND Study. *American Journal of Respiratory and Critical Care Medicine*. 2017;195(6). doi:10.1164/rccm.201602-0320OC

10. Mukhopadhyay A, Tai BC, Remani D, Phua J, Cove ME, Kowitlawakul Y. Age related inverse dose relation of sedatives and analgesics in the intensive care unit. *PLOS ONE*. 2017;12(9). doi:10.1371/journal.pone.0185212

11. Bennett S, Hurford WE. When Should Sedation or Neuromuscular Blockade Be Used During Mechanical Ventilation? *Respiratory Care*. 2011;56(2). doi:10.4187/respcare.01095
